# A Strategy for Combinatorial Cavity Design in De Novo Proteins

**DOI:** 10.3390/life10020009

**Published:** 2020-01-23

**Authors:** Christina Karas, Michael Hecht

**Affiliations:** 1 Department of Molecular Biology, Princeton University, Princeton, NJ 08540, USA; christina.e.karas@gmail.com; 2 Department of Chemistry, Princeton University, Princeton, NJ 08540, USA

**Keywords:** protein design, binary patterned amino acid sequences, four helix bundle, synthetic biology, de novo

## Abstract

Protein sequence space is vast; nature uses only an infinitesimal fraction of possible sequences to sustain life. Are there solutions to biological problems other than those provided by nature? Can we create artificial proteins that sustain life? To investigate these questions, we have created combinatorial collections, or libraries, of novel sequences with no homology to those found in living organisms. Previously designed libraries contained numerous functional proteins. However, they often formed dynamic, rather than well-ordered structures, which complicated structural and mechanistic characterization. To address this challenge, we describe the development of new libraries based on the de novo protein S-824, a 4-helix bundle with a very stable 3-dimensional structure. Distinct from previous libraries, we targeted variability to a specific region of the protein, seeking to create potential functional sites. By characterizing variant proteins from this library, we demonstrate that the S-824 scaffold tolerates diverse amino acid substitutions in a putative cavity, including buried polar residues suitable for catalysis. We designed and created a DNA library encoding 1.7 × 10^6^ unique protein sequences. This new library of stable de novo α-helical proteins is well suited for screens and selections for a range of functional activities in vitro and in vivo.

## 1. Introduction

Proteins perform myriad functions in living systems. They catalyze reactions, transmit extracellular signals, transport molecules across membranes, act as chemical messengers, and maintain cell structure. These (and other) life-sustaining functions arose in response to billions of years of selection for survival. Can functions capable of sustaining life also be found among novel proteins that did not evolve in living systems?

The collection of sequences that did not evolve in nature is vast. Even for a relatively short protein of 100 amino acids, there are 20^100^ possibilities. If we made one molecule of each, the total volume of this collection would exceed that of the known universe [1]. Thus, the overwhelming majority of possible amino acid sequences were never subjected to selective pressure in living systems. Indeed, all life on Earth survives using an infinitesimal fraction of possible sequences.

To investigate uncharted regions of sequence space, novel proteins can be produced through a number of different approaches. Recent years have seen spectacular advances in computational protein design, using ab initio and knowledge-based energy functions to specify each atom in a desired 3-dimensional structure [2,3]. At the other extreme, enormous progress has also been made producing vast combinatorial libraries of random sequences suitable for Darwinian type selections [4,5].

Our laboratory pioneered a hybrid approach that uses some level of rational design to devise libraries of semi-random sequences suitable for screens and selections in vitro and in vivo. We design the binary patterning of polar and nonpolar amino acids to specify the exposed and buried residues of a desired structure, respectively [6,7]. Thus, to design 4-helix bundles, we use a pattern of polar and nonpolar amino acids residues consistent with the formation of four amphipathic α-helices. While the pattern of polar and nonpolar residues is specified explicitly, we do not design specific sequences; instead we allow the exact identities of the polar and nonpolar residues at each position to vary combinatorically.

We have constructed and characterized several libraries of de novo α-helical proteins [6,7,8,9]. Crystal and solution structures of proteins from these libraries confirm their structures as 4-helix bundles [10,11,12]. Proteins from these libraries bind metals and cofactors [13,14], and catalyze simple reactions in vitro [15]. For future goals in synthetic biology, the most significant findings showed that several of the binary patterned proteins provide life-sustaining functions in vivo. One protein enables *E. coli* to resist otherwise toxic concentrations of copper [16], while others rescue auxotrophic cells harboring deletions of conditionally essential genes [9,17,18]. Recently, we described a novel protein that supports cell growth by functioning as a bona fide enzyme, both in vitro and in vivo [19].

The binary patterned α-helical proteins that were active in vivo formed dynamic structures that fluctuated between monomeric and dimeric forms [20]. Consequently, the high-resolution structures of these functional proteins were not determined, and their mechanisms of action could not be elucidated. Thus, our previous libraries of proteins can be binned into two classes: (i) stable structures with no function in vivo, and (ii) functional proteins that form dynamic meta-stable structures. The current work describes the construction of new libraries designed to encode the best of both: Functional proteins in the context of a well folded and stable 3-dimensional structure.

Toward this goal, we chose one of our most stable de novo proteins to serve a structural template. We then used combinatorial methods to create a new collection of proteins with diverse combinations of side chains incorporated around round a putative cavity buried in this stable structure. The goal of this approach is to develop a library of novel sequences with the capacity for function, but to do so in the context of a template that will remain sufficiently well-ordered for structure determination.

This new library combines both rational design and combinatorial diversification by introducing variability only at positions where it is likely to be beneficial. Specifically, the “top” of the bundle is targeted for the creation of a cavity and potential active site. In order to determine which residues are tolerated at each variable position, we performed a series of targeted mutagenesis studies. Results from these mutational studies, coupled with pre-existing knowledge of the catalytic propensities [21] and loop forming propensities [22] of amino acid side chains, guided the design and construction of a new library genes encoding 1.7 × 10^6^ α-helical proteins suitable for high throughput screens and selections.

## 2. Materials and Methods

### 2.1. Generation of Single Mutants

Standard site-directed mutagenesis procedures were used to create point mutants. The resulting DNA was transformed into DH5α *E. coli* and plated on LB with the appropriate antibiotic(s). Single colonies were chosen for liquid culture, plasmid extraction, and Sanger sequencing (GENEWIZ, South Plainfield, NJ, USA). The 5x-Ala mutant DNA was constructed by iterating this process.

### 2.2. Protein Purification

A single colony of *E. coli* bearing a p3GLAR plasmid containing the appropriate de novo gene was inoculated into 5 mL LB and grown for 12 h at 37 °C. The culture was diluted into 1 L LB and grown to an optical density of 0.5–0.6 at 600 nm. For induction, IPTG was added to 100 μM and the culture was incubated for 12 h at 18 °C. Cells were pelleted by centrifugation at 6000× *g* for 30 min at 4 °C. The supernatant was removed, and the cells were resuspended in ~30 mL phosphate buffer (50 mM sodium phosphate, 200 mM NaCl, pH 7.2). Cells were lysed on an Emulsiflex and debris was pelleted at 10,000× *g* for 30 min at 4 °C. The supernatant was filtered through 0.22 μM and protein was purified via Ni^2+^ sepharose resin and elution with imidazole on an ÄKTA FPLC. Fractions containing the protein of interest were pooled and subjected to size-exclusion chromatography on a HiLoad 26/600 Superdex column (GE Healthcare, Chicago, IL, USA).

### 2.3. Protein Characterization

Circular dichroism (CD) spectra of purified proteins were measured at room temperature in a quartz cuvette with a 1 mm path length. Spectra were measured with 5 scans from 200 to 250 nm. Mean residue ellipticity was calculated using protein concentrations estimated from the absorbance at 280 nm alongside sequence-based molar absorbance estimates from Protein Calculator v3.4 [23]. For thermal denaturation, absorbance at 222 nm was followed from 5 °C to 94 °C for unfolding and 94 °C to 5 °C for refolding. NMR spectra were recorded on a Bruker 800 MHz spectrometer using purified protein samples diluted in 10% D_2_O. Melting temperatures were calculated as the temperature at which CD signal was 50% of the baseline prior to unfolding.

### 2.4. Site-Saturation Mutagenesis

Primers bearing NNK codons at each of the desired positions were designed. PCR was carried out according to a standard site-directed mutagenesis protocol. The resulting DNA was transformed into *E. coli* and plated on LB + chloramphenicol or LB + ampicillin to select for the stability of the fusion protein. The resulting colonies were miniprepped and sequenced to discern which amino acid was inserted at the site of NNK mutagenesis.

### 2.5. Library Design

A library templated on S-824 was created at the DNA level by assembly of designed oligonucleotides [24]. Catalytic/core regions (CatCor) were designated NDT codon which allows for the amino acids Phe, Leu, Ile, Val, Ser, Tyr, His, Asn, Asp, Cys, Arg, and Gly. Loop forming regions (LpFor) were designated VRC codon, which allows for the amino acids: Ser, His, Asn, Asp, Arg, and Gly.

### 2.6. Oligonucleotide Assembly of De Novo Genes

For polymerase cycling assembly (PCA) reactions, 2 degenerate and 4 nondegenerate oligonucleotides were diluted to 10 μM and combined to form an equimolar mixture. The reaction mixture consisted of 4 μL of this oligonucleotide mix, 5 μL KOD polymerase buffer, 5 μL dNTPs, 3.5 μL MgSO_4_, and 1 μL of KOD polymerase in a total volume of 50 μL. For PCA reactions with flanking primer, they were added in tenfold molar ratio to the other oligonucleotides. PCA proceeded for 30 s at 95 °C, 30 s at 50 °C, and 1 min at 72 °C for 20 cycles.

For subsequent PCR reactions, 1 μL PCA product, 2 μL each of forward and reverse flanking primers (5 μM), 5 μL dNTPs, 3.5 μL MgSO_4_, and 1 μL of KOD polymerase were combined in a total volume of 50 μL. PCR was performed with an initial denaturation of 2 min at 95 °C followed by 25 cycles of 95 °C for 30 s, 50 °C for 30 s, and 72 °C for 1 min, and a final extension at 72 °C for 5 min. PCA/PCR products were analyzed by agarose gel electrophoresis on 2% agarose. For cloning into a suitable vector (p3GLAR), PCR products were purified by QIAquick PCR Purification Kit (QIAGEN, Hilden, Germany).

### 2.7. Expression Vector Assembly by Non-Restriction Cloning

De novo genes were introduced into a suitable vector (p3GLAR) by NEBuilder HiFi DNA Assembly. Briefly, vectors were linearized by PCR and purified by QIAquick PCR Purification Kit (QIAGEN). 100 ng of vector was combined with 2-fold molar excess of inserts and 10 μL master mix in a total volume of 20 μL. Samples were incubated at 50 °C for 30 min and stored at −20 °C until transformation. 2 μL of ligation mixture was transformed into 50 μL electrocompetent DH5α *E. coli*. 1 mL SOC medium was added before outgrowth for 1 h at 37 °C, 200 rpm. Cells were plated on the appropriate selective medium. Individual clones were sequenced to assess the accuracy and quality of library assembly.

### 2.8. Transformation and Batch Purification of Plasmid DNA

In total, 250 μL of DH5α electrocompetent cells were combined with 5 μL assembled library DNA and transferred to a 2 mm electroporation cuvette. Following electroporation, cells were resuspended in 5 mL SOC and allowed to recover for 1 h at 37 °C with shaking at 200 rpm. 1 mL of this undiluted mixture was plated onto each large, square petri dish of LB + Amp and allowed to dry. The plate was incubated overnight at 37 °C. On the following day, the plate was divided into eighths and one section was counted; this was multiplied by 8 to give the total CFU count per plate. 20 mL of LB media was added to the plate and it was placed on the shaking incubator to gently resuspend cells. Cell density was quantified by OD_600_.

DNA was then purified by midiprep. The known cell density allows adequate loading so that the DNA yield from these cells does not exceed the binding capacity of the column. This process was repeated until a total of ~10^6^ CFU had been isolated. The ~35 total midipreps were combined, accounting for differences in their DNA concentration.

### 2.9. β-Lactamase Assay

To adapt this assay for use in our system, we obtained the gBlock for TEM-1 β-lactamase and cloned it into p3GLAR, a modified pCA24N plasmid. Next, we created the fusion proteins by standard restriction cloning. Once the sequences were verified by Sanger sequencing, *E. coli* bearing these constructs were grown in LB with chloramphenicol as a selectable marker for the plasmid. After overnight growth, the culture was diluted 1:1000 and incubated at 37 °C with shaking. Once the cultures reached mid-log phase (OD_600_ = ~0.5), the optical density was normalized in phosphate-buffered saline solution to ensure an equal number of cells in each sample. A tenfold serial dilution was made in 96-well plates and the resulting cell solutions were pipetted onto petri dishes containing various concentrations of ampicillin and a constant 100 μM IPTG. The resulting plates should contain spots with decreasing amounts of cell growth due to the dilution used. Each row represents one construct, while each column represents a tenfold dilution relative to the preceding column. Growth of the more dilute samples indicates greater resistance to ampicillin, and therefore a more stable protein of interest. Sensitivity to ampicillin would present as poor growth of the diluted samples and could indicate an erroneous or poorly-folded construct.

### 2.10. Next-Generation Sequencing

The quality and diversity of the library was assessed by high-throughput sequencing (Genomics Core Facility, Lewis-Sigler Institute for Integrative Genomics, Princeton University). Briefly, PCR was performed on plasmid DNA to generate amplicons for sequencing (MiSeq Micro, read length 300 nt). The chosen amplicon covered all the variable regions to retain information for complete individual sequences. These data were translated to the corresponding amino acids and analyzed in depth for each of the variable positions, and a constant position as a control. From these data, the percent occurrence of each amino acid at a particular position was determined.

## 3. Results

### 3.1. Protein S-824 Tolerates the Deletion of Side-Chains in Its Hydrophobic Core

The overall goal of this work is to design and produce a combinatorial library of cavities—which may contain active sites—into the context of a stable de novo protein structure. Toward this goal, we sought a structurally-characterized and highly stable protein from our binary patterned library of 4-helix bundles. We chose protein S-824, originally isolated from a 2nd generation library of sequences. The experimentally determined structure of S-824 reveals a well-ordered 4-helix bundle, with a fully hydrophobic core, in accordance with the binary patterned design [11]. Thermodynamic studies demonstrated that S-824 is extremely stable to chemical and thermal denaturation, with a T_m_ near the boiling point of water [7].

**Figure 1:**
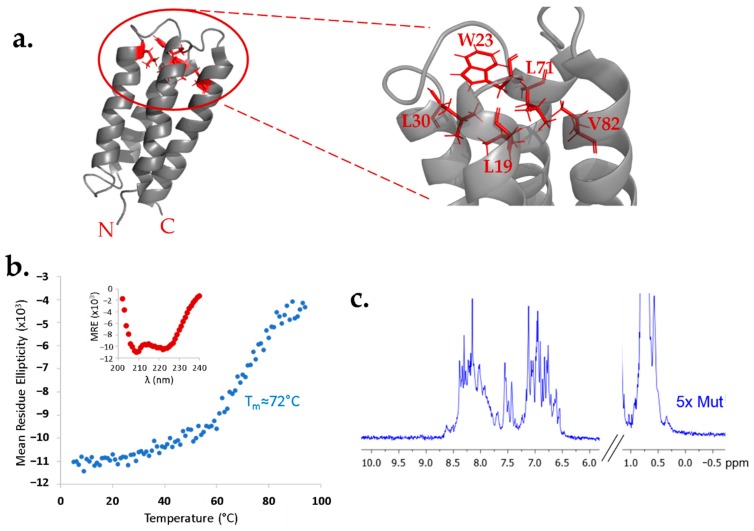
Characterization S-824 5x-Ala mutant. (**a**) Structure of S-824 showing the hydrophobic sidechains (red) eliminated in this mutant. (**b**) Inset: CD spectrum of S-824 5x-Ala containing minima at 208 and 222 nm, indicative of α-helical secondary structure. Blue points: Thermal denaturation of 5x-Ala reveals cooperative denaturation with a midpoint near 72 °C. (**c**) ^1^H NMR spectrum of the 5× mutant.

To test whether the structure of protein S-824 can tolerate a buried cavity, we replaced five buried side chains near the “top” of the bundle with five alanines. In this 5x-Ala mutant, five large hydrophobic side chains (Leu19, Trp23, Leu30, Leu71, Val82) were simultaneously reduced to single methyl groups (Figure 1a). The 5-fold mutated protein was compared to protein S-824 by circular dichroism (CD) and nuclear magnetic resonance (NMR) spectroscopy (Figure 1b,c). The CD spectrum of 5x-Ala (Figure 1b; inset) shows an α-helical secondary structure. Thermal denaturation of the mutant protein revealed that, although the melting point was somewhat reduced relative to the parental S-824 protein, 5x-Ala is very stable, with a T_m_ close to 72 °C.

The ^1^H NMR spectrum of 5x-Ala (Figure 1c) shows reasonable chemical shift dispersion, consistent with a stably folded 3-dimensional structure. Although the dispersion is somewhat diminished relative to the extremely sharp spectrum of S-824 [7,11], the spectrum of 5x-Ala is considerably better dispersed than that of the dynamic binary patterned protein SynGltA, which was shown previously to restore the growth of *ΔgltA E. coli* under nutrient-limiting conditions [9]. In contrast to the dynamic SynGltA protein, 5x-Ala appears to form a well-ordered structure. These results suggest the structure of protein S-824 is robust and may be stable enough to tolerate a cavity in its hydrophobic core.

**Figure 2:**
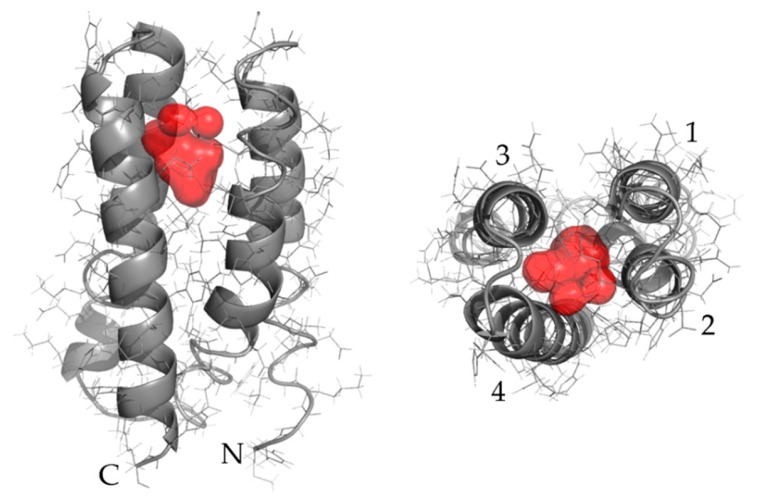
Removal of hydrophobic sidechains from the buried core of S-824 results in an apparent cavity. The sequence of 5x-Ala (L19A, L30A, W23A, L71A, V82A) was submitted to I-TASSER for structure prediction, excluding templates derived from binary patterned libraries. The top model for 5x-Ala (gray) was analyzed in PyMol and a cavity (red) was detected. Depicted here are the side view (left) with N- and C- termini labeled and the top view (right) with helices labeled 1 through 4.

To further investigate this hypothesis, structure prediction was performed on the sequence of 5x-Ala. I-TASSER [25] revealed that the top model contained a cavity (Figure 2). Novel proteins derived from this variant may bind small molecules in this putative cavity.

### 3.2. Protein S-824 Tolerates a Wide Range of Polar and Nonpolar Side-Chains in Its Hydrophobic Core

The results described in the previous section show that the core of protein S-824 tolerates the removal of five large hydrophobic side chains. Thus, S-824 is structurally permissive. However, alanine side chains rarely play important roles in active sites [21]. Therefore, we sought to assess whether the proposed cavity would also be chemically permissive. Toward this goal, we substituted the full range of polar and nonpolar residues in positions around the planned cavity. This was accomplished by performing site-saturation mutagenesis on each of the five positions. Starting with the 5x-Ala sequence, we used NNK degenerate primers for PCR-based mutagenesis. (N = A, G, C or T and K = G or T.)

**Figure 3:**
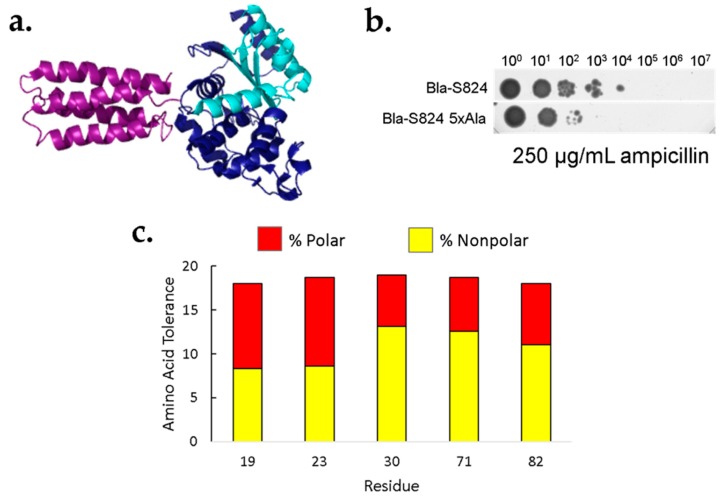
Most amino acids are tolerated at five core positions of S-824. (**a**) To link in vivo protein stability to antibiotic resistance, library proteins (purple) were fused to β-lactamase between the N- and C-terminal domains (light and dark blue). (**b**) Tenfold dilutions of cells expressing fusions β-lactamase were plated on 250 μg/mL ampicillin. The top row shows cells expressing a β-lactamase fusion to the parental protein, S824, while the bottom row shows cells expressing a fusion to the 5x-Ala mutant. (**c**) Following site-saturation mutagenesis and selection with β-lactamase, colonies were sequenced. Bar height: number of amino acids tolerated out of a possible 20 at each of the core positions. Red: hydrophilic residues; yellow: hydrophobic residues.

Saturation mutagenesis of these five sites would yield 95 (i.e., 5 × 19) singly substituted proteins. Rather than purifying all these proteins for individual characterization, we used an in vivo screen for protein stability. In this screen, a protein of interest (POI) is inserted into the middle of the β-lactamase sequence [26], as depicted in Figure 3a. Since β-lactamase functions by degrading β-lactam antibiotics, this fusion couples in vivo protein stability to antibiotic resistance. In cases where the POI folds into a stable structure, the function of the neighboring β-lactamase domains is intact, leading to antibiotic resistance. Conversely, in cases where the POI is unfolded, proteolytic degradation is likely to sever the linkage between the N- and C-terminal domains, leading to domain dissociation, and antibiotic sensitivity. Because cells expressing β-lactamase are resistant to ampicillin (and related antibiotics), well-folded POIs can be selected from libraries of poorly folded sequences. Moreover, because higher levels of correctly folded β-lactamase enable resistance to higher levels of ampicillin, the stringency of the selection can be tuned as needed.

We tested the efficacy of the β-lactamase assay by comparing the known stability of the parental S-824 protein to that of the 5x-Ala mutant. Serial dilutions of cells expressing either S-824 or 5x-Ala were spotted onto a plate containing 250 μg/mL ampicillin. As shown in Figure 3b, the β-lactamase assay shows that the 5x-Ala mutant is less stable than the original S-824 protein, thereby recapitulating the stability difference measured by following the denaturation of the purified proteins by CD spectroscopy (Figure 1b). Further experiments validating the β-lactamase assay are shown in the Supplementary Information, Figure S1.

Next, our collections of NNK mutants were tested in the β-lactamase assay. For this assay, we assessed growth on 40 μg/mL ampicillin. This concentration was chosen because insertions of either S-824 or the 5x-Ala mutant into β-lactamase enable growth, but insertion of the poorly-folded SynGltA does not. Following plating on ampicillin, the resulting colonies were restreaked and sequenced to determine which amino acids were present among the surviving colonies.

The results of this assay suggest that in the context of the 5x-Ala cavity, a wide variety of chemically diverse amino acids is tolerated at each of the five positions (Figure 3c). For example, at position 19, the selected sequences contained all amino acids except phenylalanine and proline. At position 23, all residues except lysine were present in the sequenced population of ampicillin resistant colonies. It is important to note that the tolerances shown in Figure 3c represent minimal estimates. At each of the five positions we sequenced approximately 50 ampicillin resistant colonies. It is possible that all 20 amino acids would lead to ampicillin resistance, but some were not seen in our limited data set. Irrespective of the exact number of amino acids seen at each position, the data shown in Figure 3c make it clear that each of these five positions tolerates a wide range of polar and nonpolar amino acids. More information about the prevalence of specific amino acids within the ampicillin-resistant population is shown in Figure S2.

Altogether, our findings with the 5x-Ala mutant and site saturation mutagenesis at these five residues suggest that many (perhaps all) amino acid side chains are tolerated at the putative cavity site of protein S-824. Therefore, we chose this protein as the structural template for the design of a combinatorial library of buried cavities.

### 3.3. Design and Construction of a Cavity Library into the Structural Template of Protein S-824

**Figure 4:**
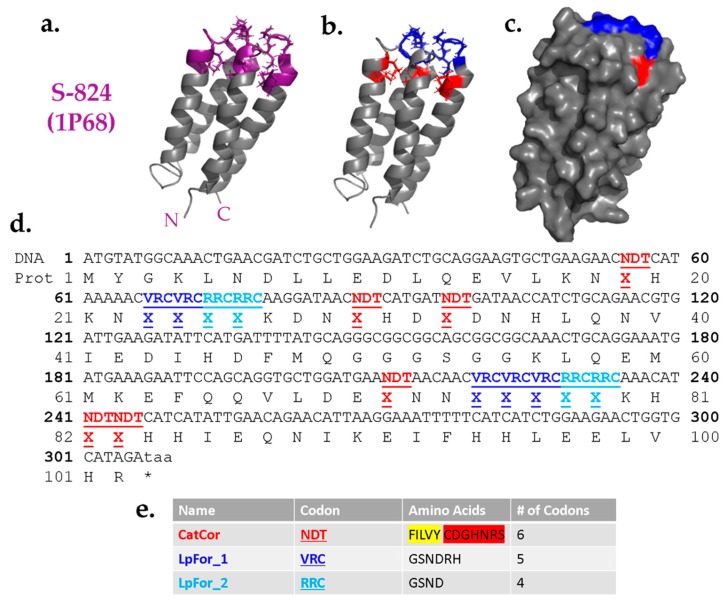
Design of a combinatorial library templated on protein S-824. (**a**) NMR structure of S-824. Purple: proposed variable region; Gray: invariant region. N- and C-termini labeled. (**b**) Specifics of library design. Red: catalytic/core residues (CatCor) encoded by NDT codon; blue: loop-forming regions (LpFor) encoded by VRC codon. (**c**) Surface view. CatCor residues are mostly buried hydrophobic residues while LpFor are surface-exposed residues found in the loops. (**d**) DNA and protein sequence of the novel genes. A mixture of amino acids is designated by X. (**e**) Degenerate codon and amino acids selected for each variable position. For CatCor, hydrophobic residues are highlighted in yellow and polar residues are highlighted in red.

Guided by the results described above, we designed a combinatorial library of cavity mutants into the structure of protein S-824. The proposed cavities were designed to sit near the “top” of the bundle, such that side chains projecting toward the cavity could originate from any of the four α-helices, or either of two inter-helical loops (Figure 4a). Based on their locations, the positions around the proposed cavity were divided into two classes: Catalytic/Core residues and Loop-Forming residues (Figure 4b); the former are buried positions while the latter tend to be more surface-exposed (Figure 4c).

As the name implies, the Catalytic/Core (CatCor) residues were chosen from a mixture of side chains that can serve either as catalytic residues or contribute to packing of the hydrophobic core. These residues included Ser, Tyr, His, Asn, Asp, Cys, Arg, Phe, Leu, Ile, Val, and Gly (Figure 4e). However, to avoid burying too many charges, the 12 residues in the CatCor set were not represented equally. Instead, this set was designed to be ~61% hydrophobic. The remaining 39% comprise residues with a high catalytic propensity (plus glycine). This breakdown was chosen because too high a proportion of polar residues would increase the chance of encoding proteins with multiple like charges in the putative cavity, a feature likely to destabilize the structure. Among the subset of hydrophobic residues, Phe was included less frequently than Leu, Ile, and Val due to its larger size. By minimizing the occurrence of the bulkiest hydrophobic side chains, we sought to avoid overcrowding the putative cavity. Additionally, to avoid complications due to disulfide bonds, cysteine was encoded as 0.75% of the mix. The 12 CatCor residues can be encoded by the degenerate DNA codon NDT. (N = A, G, C, T and D = A, G, T.) To favor the desired percentages of CatCor residues, the bases at the degenerate positions N and D were not added as equimolar mixtures. Instead, the percentages of each base chosen to yield the desired percentages of amino acids (see Methods). More detailed information about the prevalence of each individual amino acid within the CatCor codon is provided in the Supplementary Information (Figure S3).

The Loop-Forming (LpFor) residues were chosen from a mixture of polar side chains that are suitable in loops and also found frequently in active sites. Thus, the set of LpFor residues was designed to be 74% Gly and Ser, with the remaining 26% containing His, Asn, Asp, and Arg. This mixture of amino acids was encoded by the degenerate DNA codon VRC (V = A, G, C and R = A, G.) As above, to ensure the desired percentages, the degenerate nucleotides were not used in equimolar ratios (see Methods). For LpFor_2 positions, the set of possible amino acids was further restricted to an RRC codon encoding only Gly, Ser, His, and Asn, with an increased probability of flexible amino acids to facilitate loop formation. The entire DNA sequence of the library of de novo genes is depicted in Figure 4d.

To encode the designed library of proteins, we constructed the corresponding library of synthetic genes. Oligonucleotides with regions of degeneracy corresponding to the proposed variable regions were ordered from IDT and assembled into double-stranded de novo genes by polymerase cycling assembly (PCA) and PCR [24]. The number of PCA and PCR cycles were optimized to yield the greatest yield and diversity of correct sequences [27]. NEBuilder HiFi DNA Assembly was performed to introduce the de novo genes into a suitable vector for expression in *E. coli*. To generate the full library, assembled plasmids were transformed into electrocompetent DH5α cells and plated on large square petri dishes. At this stage, each colony should contain cells bearing a different de novo gene. Colonies were counted and the process was repeated until a total of >10^6^ independent colonies had been isolated. Next, the colonies were scraped together, resuspended in buffer and diluted to a suitable optical density for plasmid midipreps. Accounting for differences in DNA concentration, plasmid midipreps were combined to create the full library of genes.

The theoretical diversity of the library can be calculated as the product of the number of possibilities at each variable position—the number of possible amino acids included in the set raised to the total number of positions. For CatCor (12 amino acids, 6 codons), LpFor_1 (6 amino acids, 5 codons), and LpFor_2 (4 amino acids, 4 codons), the calculation is as follows: 12^6^ ∙ 6^5^ ∙ 4^4^ = 5.94 × 10^12^ possible sequences. This theoretical diversity is larger than can be constructed or assayed experimentally. Hence, the actual library of de novo sequences described here is not the complete collection of all possible variants at the chosen locations, but rather a sampling of novel sequences amidst this previously unexplored region of sequence space.

### 3.4. Characterization of the Library of Genes

Once the library was generated, we asked several questions: Are the sequences correct? Are they expressed? Are the resulting proteins stable?

To address the first question, colonies were picked randomly and plasmids were purified and sequenced. Since the coupling efficiency of DNA synthesis is <100% per base, libraries of synthetic genes inevitably contain errors. For our library, DNA sequencing revealed that ~70% were correct on-library sequences, lacking frameshifts or stop codons. Although erroneous sequences can be weeded out using genetic pre-selections for uninterrupted genes [8], we decided that a library containing 70% correct sequences was acceptable and would serve well as the input for future screens and selections for function.

**Figure 5:**
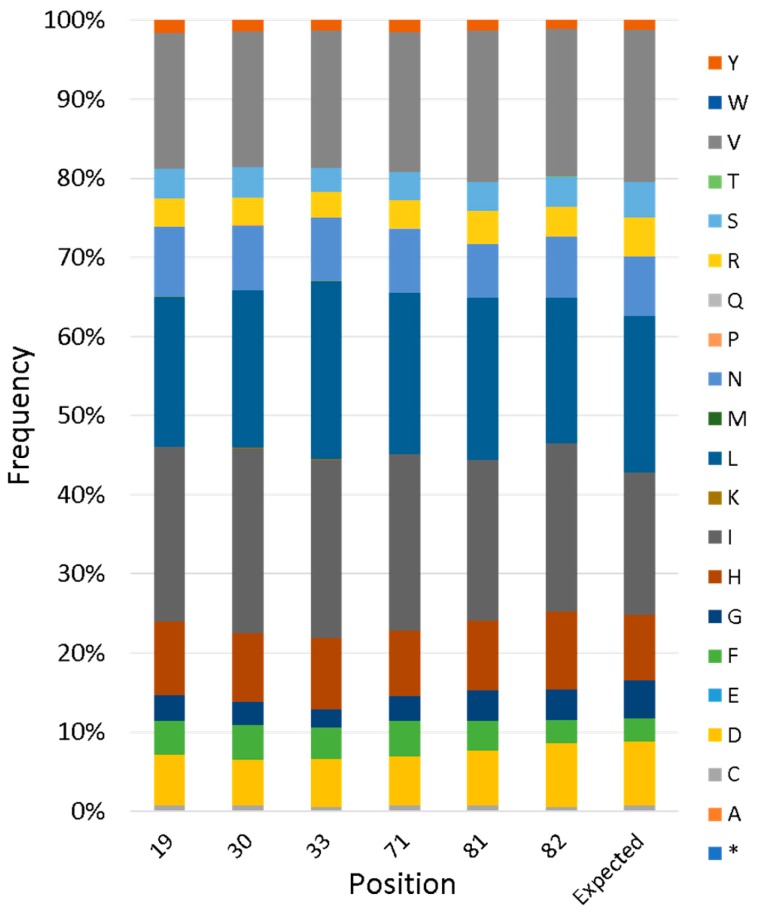
Next-generation sequencing of a combinatorial library of cavities. Plasmid DNA from the library was subjected to PCR to create amplicons spanning both variable regions. These amplicons were sequenced on MiSeq Micro and analyzed for the encoded amino acids at each variable position. Bar height represents the frequency of a given amino acid; the rightmost bar indicates the expected amino acid composition for CatCor positions according to the library design. Asterisk indicates a stop codon.

To evaluate the actual diversity of the full library, next-generation sequencing (NGS) was performed. This revealed that the library contains 1,700,000 unique sequences, two-thirds of which are on-library (i.e., without frameshifts or stop codons). For variable positions, the frequency of each amino acid agreed closely with the designed input, as described in the previous section (Figure 5).

### 3.5. Characterization of the Library of Synthetic Proteins

To estimate the fraction of proteins that are stably folded, we cloned the collection of genes into the β-lactamase folding reporter (Figure 3a). Transformants were plated on two types of selective media: (i) chloramphenicol (CAM), or (ii) 40 µg/mL ampicillin + 100 µM IPTG (AMP + IPTG). The CAM plates selected total transformants, while the AMP + IPTG plates induced expression and selected proteins that are stably folded.

Sequencing colonies from the CAM plate confirmed that, as above, ~70% were correct on-library sequences. The remaining 30% contained frameshifts or empty vectors. The number of colonies on the AMP + IPTG plates was 72.6% of that on the CAM plates. Thus, the fraction of the library that survived the folding reporter selection is nearly identical to the fraction of the library containing correct sequences on the non-selective plates. This suggests that nearly all the error-free sequences in the library are relatively stable. While the β-lactamase folding reporter does not produce an exact readout of thermodynamic stability, our finding that nearly all the library proteins are more stable than the dynamic functional protein SynGltA (see above) provides initial evidence that the goal of producing a library of >1 million sequences on a structurally stable scaffold was successful.

### 3.6. Biophysical Characterization of Individual Proteins Reveals Stably Folded Structures

**Figure 6:**
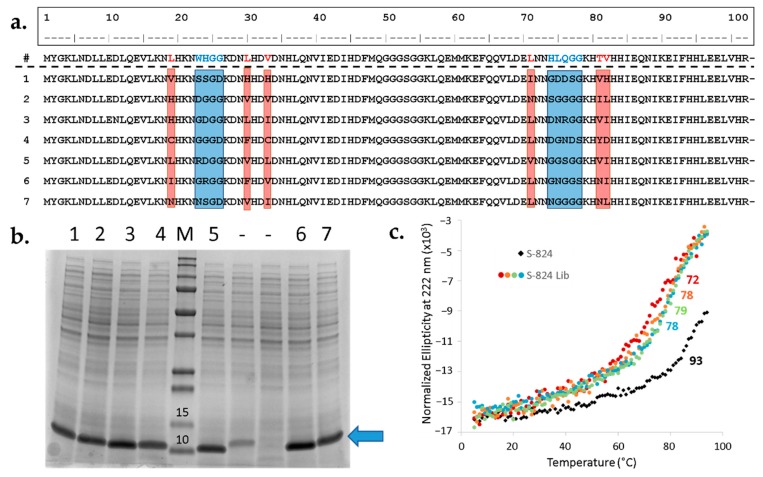
Randomly picked library members agree with design, express well, and are α-helical. (**a**) Sequence alignment demonstrating diversity of mutated sites. First line: Amino acid sequence of S-824, the parental protein for library generation. Positions subjected to semi-randomization are in red (CatCor) and blue (LpFor). Below are seven well-expressed sequences from the library with variable regions highlighted. (**b**) The majority of arbitrarily chosen library members express well. Library DNA was transformed into BL21 (DE3) *E. coli* and nine colonies were picked from the resulting plate for small-scale overexpression studies. Total protein content was assessed by SDS-PAGE. M: molecular weight marker with relevant values (kDa) listed above the bands, Precision Plus Protein™ All Blue Protein Standards (Bio-Rad); 1–7: library proteins yielding good expression; (-): off-library sequences yielding reduced or no protein of the expected size. Blue arrow indicates expected molecular weight of library proteins. (**c**) Thermal denaturation of S-824 and randomly chosen library proteins. The library proteins (colored circles) undergo cooperative denaturation with midpoints ranging from 72 °C to 78 °C (listed beside the curves). This is a slight reduction in T_m_ relative to the parental S-824 (black diamonds).

After the library DNA was demonstrated to be remarkably consistent with the design, the next step was to investigate the expression, stability, and foldedness of the new proteins. To that end, the library was transformed into BL21 (DE3) and nine sequences were arbitrarily chosen for characterization. First, cells were cultured in liquid medium in the presence of IPTG inducer and the resulting undiluted whole-cell samples were analyzed for protein content via SDS-PAGE (Figure 6b). Seven out of nine unselected sequences displayed a prominent band at the expected molecular weight, suggesting the majority of library expresses well. Next, the sequences of the strong expressers were determined (Figure 6a). The resulting alignment demonstrates diverse amino acid composition within the designed variable regions, but not elsewhere in the protein. Moreover, as specified by the design, most of the sequences contain one or several polar side chains in the putative cavity.

Previous studies demonstrated that most proteins in our binary patterned libraries bind to Ni^2+^ resins [20,28]. This is presumably due to the abundance of surface-exposed histidines encoded by the binary code. Since the new library only introduces variation in a targeted region, many surface-exposed histidines are present in the constant regions. This enabled rapid purification of these seven proteins using immobilized metal ion chromatography (IMAC) without the need for an added His-tag [10,20,28]. Following IMAC, the seven proteins were further purified by size exclusion chromatography (SEC). The purified proteins displayed circular dichroism (CD) spectra characteristic of α-helical structure, with minima at 208 and 222 nm. For all seven proteins, thermal denaturation occurred cooperatively between 72 °C and 78 °C (Figure 6c). Although these T_m_ values are lower than that of S-824, these proteins have thermal stabilities similar to or higher than most mesophilic natural proteins [29].

Overall, these results demonstrate the successful production of a library of >10^6^ novel proteins with the potential for active site cavities incorporated into the structurally stable fold of a novel 4-helix bundle.

## 4. Discussion

The number of protein sequences that are possible vastly exceeds the number of atoms in the universe [1]. Consequently, despite billions of years of evolutionary “experiments” in countless numbers of cells, nature has sampled only a miniscule fraction of what is possible. Moreover, because (i) all life on earth descended from common ancestry, and (ii) new proteins arise by mutational variation of pre-existing sequences, nature’s exploration of sequence space has been limited to neighborhoods around a restricted number of ancestral progenitors.

The enormous number of possible sequences relative to the relatively small number of sequences that actually sustain life on Earth led us to ask whether alternative proteins—unrelated to those in current or historical biospheres—might also be capable of sustaining living organisms.

To address this question, we have spent three decades designing and testing combinatorial libraries of novel proteins. To favor stably folded structures, we developed a strategy that uses binary patterning of polar and nonpolar amino acids to favor folding into structures with uniformly hydrophobic interiors and completely polar surfaces. This binary code strategy succeeded in producing well-folded and highly stable α-helical proteins [7]. Some of these novel proteins bound metals, cofactors, and other small molecules, and in some cases, the novel proteins catalyzed reactions in vitro [14,15].

To assess whether these alternative sequences might also be capable of sustaining life, we searched our libraries for proteins that can rescue the deletion of natural proteins that are essential for life. Ultimately, we isolated novel sequences that rescued the deletions of *∆serB*, *∆gltA*, *∆ilvA*, and *∆fes*, which code for phosphoserine phosphatase, citrate synthase, threonine deaminase, and enterobactin esterase, respectively [9]. Several of these novel proteins achieved rescue by rewiring gene regulation and/or up-regulating endogenous and promiscuous *E. coli* proteins [17,18]. Thus far, however, only one of the de novo proteins is a bona fide enzyme. This protein, called Syn-F4, rescues *∆fes.* Syn-F4 is the first example of a protein that is not derived from any natural sequence, but nonetheless provides a life-sustaining enzymatic activity [19].

Perhaps it is not surprising that life-sustaining enzymatically active proteins occurred only rarely in our binary patterned libraries. After all, the binary code strategy was devised to produce libraries of thermodynamically stable structures, with no consideration of function. Thus, all polar residues were designed to be exposed to solvent, while the buried parts of these structures were designed to include only nonpolar residues. This extremist strategy favors protein stability; however, it is not well-suited for producing active site cavities incorporating buried, or partially buried, polar side chains. (Of course, nature faced a similar conundrum in selecting proteins that are stabilized by hydrophobic cores, while simultaneously including polar/catalytic residues into active sites. Nature has spent billions of years solving this problem; >10^8^ fold more time than protein designers.)

The approach chosen in this study differs from how a buried cavity might have arisen in early proteins. Ohno’s dilemma states that a gene product must have a useful function at all stages of its evolution to be acted upon by positive selection [30]. Our study circumvents Ohno’s dilemma by introducing multiple simultaneous mutations that are likely to yield a cavity. The library presented in this study represents a search for alternative sequences that may sustain life but are not necessarily representative of ancient natural sequences.

Although de novo protein design has advanced remarkably in recent years [2], the ability to create functional proteins from scratch or incorporate function to existing de novo proteins is still expanding [31]. Recent work from the Baker lab used computational methods to design β-bulge proteins with large cavities well-suited to ligand binding [32]. This powerful method involves creating small numbers of high-quality proteins geared towards a specific structure and function. Custom designing backbones for the reaction of interest allows researchers to overcome the limitations of previous studies, such as the unpredictable structural changes that often occur when introducing an idealized active site to a preexisting structure. Our method presented in the current work is a complementary approach involving making millions of novel proteins with diverse functional potential. The advantage of our method is that it makes relatively few assumptions about the types of reactions that will be catalyzed and thus does not need to be repeated to assess each new function.

The current work aims to modify the original binary code strategy by allowing occasional polar residues into buried (or partially buried) cavities. Because such burial will inevitably decrease the thermodynamic stability of the resulting protein, we pursued two strategies to ameliorate the destabilizing impact: First, we chose to start with an extremely stable template protein, which would tolerate some level of destabilization. Second, we did not design a specific polar cavity; instead we allowed for the possibility that some polar residues will be tolerated at some positions better than others, and therefore designed a combinatorial library of alternative cavities.

In accordance with these two considerations, we chose the extremely stable protein, S-824, as our starting template, and planned the design of >10^6^ alternative cavities into this template. Initial experiments probed whether a particular location near the “top” of the S-824 4-helix bundle would tolerate the simultaneous deletion of five large hydrophobic side chains. Results from these experiments demonstrated that Leu19, Trp23, Leu30, Leu71, Val82 could all be reduced to alanine simultaneously without destroying the structure of the helical bundle. Next, we showed that the putative cavity in this 5x-Ala mutant tolerated a wide range of residues, including polar and charged side chains. Building on these results, we developed methodologies, designed sequences, and constructed a library containing 1.7 × 10^6^ alternative cavities.

Characterization of this library demonstrated that the newly developed methodologies are robust and generate >10^6^ diverse sequences with >70% accuracy. The encoded proteins were characterized using both an in vivo screen for folding, and biophysical studies of purified proteins. Both assays showed that the vast majority of sequences formed stably folded structures.

These results demonstrate that it is possible to modify the binary code strategy in ways that enable the design of proteins that are thermodynamically stable and simultaneously incorporate polar active sites. The approaches developed here will enable the design of further libraries of buried cavities into the structures of other binary patterned de novo proteins. Specifically, ongoing work is focused on designing cavity libraries into the dimeric 4-helix bundle of the Syn-F4 enzyme. These new libraries are poised for screens and selections aimed at discovering a range of proteins that did not evolve in living systems, but which nonetheless provide activities that enable the growth of living organisms.

## Supplementary Materials

The following are available online at https://www.mdpi.com/2075-1729/10/2/9/s1, Figure S1: Validation of β-lactamase stability selection for *de novo* proteins. Figure S2. Raw data for the structural tolerance of S-824. Figure S3. Amino acid percentages for variable codons.

## References

[1] Beasley J.R., Hecht M.H. (1997). Protein design: The choice of de novo sequences. J. Biol. Chem..

[2] Huang P.S., Boyken S.E., Baker D. (2016). The coming of age of de novo protein design. Nature.

[3] Baker D. (2019). What has de novo protein design taught us about protein folding and biophysics?. Protein Sci..

[4] Keefe A.D., Szostak J.W. (2001). Functional proteins from a random-sequence library. Astrobiology.

[5] Seelig B., Szostak J.W. (2007). Selection and evolution of enzymes from a partially randomized non-catalytic scaffold. Nature.

[6] Kamtekar S., Schiffer J.M., Xiong H.Y., Babik J.M., Hecht M.H. (1993). Protein design by binary patterning of polar and nonpolar amino-acids. Science.

[7] Wei Y.N., Liu T., Sazinsky S.L., Moffet D.A., Pelczer I., Hecht M.H. (2003). Stably folded de novo proteins from a designed combinatorial library. Protein Sci..

[8] Bradley L.H., Kleiner R.E., Wang A.F., Hecht M.H., Wood D.W. (2005). An intein-based genetic selection allows the construction of a high-quality library of binary patterned de novo protein sequences. Protein Eng. Des. Sel..

[9] Fisher M.A., McKinley K.L., Bradley L.H., Viola S.R., Hecht M.H. (2011). De Novo Designed Proteins from a Library of Artificial Sequences Function in Escherichia Coli and Enable Cell Growth. PLoS ONE.

[10] Arai R., Kimura A., Kobayashi N., Matsuo K., Sato T., Wang A.F., Platt J.M., Bradley L.H., Hecht M.H. (2012). 3VJF: Crystal structure of de novo 4-helix bundle protein WA20. Worldw. Protein Data Bank.

[11] Wei Y.N., Kim S., Fela D., Baum J., Hecht M.H. (2003). Solution structure of a de novo protein from a designed combinatorial library. Proc. Natl. Acad. Sci. USA.

[12] Go A., Kim S., Baum J., Hecht M.H. (2008). Structure and dynamics of de novo proteins from a designed superfamily of 4-helix bundles. Protein Sci..

[13] Rojas N.R.L., Kamtekar S., Simons C.T., McLean J.E., Vogel K.M., Spiro T.G., Farid R.S., Hecht M.H. (1997). De novo heme proteins from designed combinatorial libraries. Protein Sci..

[14] Wang M.S., Hoegler K.J., Hecht M.H. (2019). Unevolved De Novo Proteins Have Innate Tendencies to Bind Transition Metals. Life.

[15] Patel S.C., Bradley L.H., Jinadasa S.P., Hecht M.H. (2009). Cofactor binding and enzymatic activity in an unevolved superfamily of de novo designed 4-helix bundle proteins. Protein Sci..

[16] Hoegler K.J., Hecht M.H. (2016). A de novo protein confers copper resistance in Escherichia coli. Protein Sci..

[17] Digianantonio K.M., Hecht M.H. (2016). A protein constructed de novo enables cell growth by altering gene regulation. Proc. Natl. Acad. Sci. USA.

[18] Digianantonio K.M., Korolev M., Hecht M.H. (2017). A Non-natural Protein Rescues Cells Deleted for a Key Enzyme in Central Metabolism. Acs Synth. Biol..

[19] Donnelly A.E., Murphy G.S., Digianantonio K.M., Hecht M.H. (2018). A de novo enzyme catalyzes a life-sustaining reaction in Escherichia coli. Nat. Chem. Biol..

[20] Murphy G.S., Greisman J.B., Hecht M.H. (2016). De Novo Proteins with Life-Sustaining Functions Are Structurally Dynamic. J. Mol. Biol..

[21] Bartlett G.J., Porter C.T., Borkakoti N., Thornton J.M. (2002). Analysis of catalytic residues in enzyme active sites. J. Mol. Biol..

[22] Parker M.H., Hefford M.A. (1997). A consensus residue analysis of loop and helix-capping residues in four-alpha-helical-bundle proteins. Protein Eng..

[23] Protein Calculator v3.4. http://protcalc.sourceforge.net/.

[24] Acevedo-Rocha C., Reetz M.T. (2014). Assembly of Designed Oligonucleotides: A Useful Tool in Synthetic Biology for Creating High-Quality Combinatorial DNA Libraries. Directed Evolution Library Creation.

[25] Roy A., Kucukural A., Zhang Y. (2010). I-TASSER: A unified platform for automated protein structure and function prediction. Nat. Protoc..

[26] Foit L., Morgan G.J., Kern M.J., Steimer L.R., von Hacht A.A., Titchmarsh J., Warriner S.L., Radford S.E., Bardwell J.C.A. (2009). Optimizing Protein Stability In Vivo. Mol. Cell.

[27] TerMaat J.R., Pienaar E., Whitney S.E., Mamedov T.G., Subramanian A. (2009). Gene synthesis by integrated polymerase chain assembly and PCR amplification using a high-speed thermocycler. J. Microbiol. Methods.

[28] Arai R., Kobayashi N., Kimura A., Sato T., Matsuo K., Wang A.F., Platt J.M., Bradley L.H., Hecht M.H. (2012). Domain-Swapped Dimeric Structure of a Stable and Functional De Novo Four-Helix Bundle Protein, WA20. J. Phys. Chem. B.

[29] Taylor T.J. (2010). Vaisman II: Discrimination of thermophilic and mesophilic proteins. BMC Struct. Biol..

[30] Bergthorsson U., Andersson D.I., Roth J.R. (2007). Ohno’s dilemma: Evolution of new genes under continuous selection. Proc. Natl. Acad. Sci. USA.

[31] Dawson W.M., Rhys G.G., Woolfson D.N. (2019). Towards functional de novo designed proteins. Curr. Opin. Chem. Biol..

[32] Marcos E., Basanta B., Chidyausiku T.M., Tang Y., Oberdorfer G., Liu G., Swapna G.V.T., Guan R., Silva D., Dou J. (2017). Principles for designing proteins with cavities formed by curved β sheets. Science.

