# Supplementary material for: A Strategy for Combinatorial Cavity Design in De Novo Proteins
Source: Life (Basel). 2020 Jan 23;10(2):9. doi: 10.3390/life10020009 (PMC7175167; doi:10.3390/life10020009)
Supplement: Supplementary file 1 [file life-10-00009-s001.pdf]

Supplementary Information: A Strategy for Combinatorial Cavity Design in *de novo* Proteins

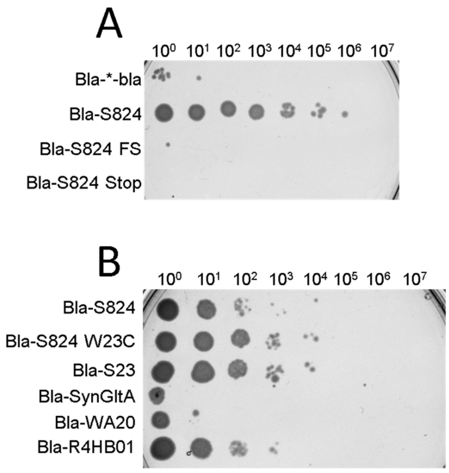

Figure S1. Validation of β-lactamase stability selection for *de novo* proteins. Serial dilutions of cells bearing various fusion constructs were plated on various concentrations of ampicillin. (**A**) Comparison of off-library versus full-length sequences at 25 µg/mL ampicillin. Bla-*-bla: parental construct sensitive to ampicillin; Bla-S-824: construct bearing a full-length, well-folded protein displays antibiotic resistance; Bla-S-824 FS: introducing a frameshift mutation restores sensitivity to ampicillin; Bla-S-824 Stop: a stop codon has a similar effect. (**B**) Comparison of well- and poorly-folded proteins at 250 µg/mL ampicillin. Bla-S-824 W23C: well-folded single mutant; Bla-S23: a protein from the same library as S-824; SynGltA: a poorly-folded protein; Bla-WA20: a protein with a stable homodimeric structure; Bla-R4HB01: a very thermostable, well-folded protein from a library designed to form right-handed 4-helix bundles.

a.

	Residue				
	19	23	30	71	82
A	3	3	2	11	4
C	4	1	3	2	5
D	3	1	2	1	1
E	3	2	0	2	4
F	0	1	3	1	2
G	3	4	8	6	7
H	8	3	3	2	0
I	1	2	4	1	2
K	3	0	1	0	0
L	1	6	12	6	7
M	4	3	3	2	3
N	1	4	3	1	1
P	0	3	3	5	3
Q	1	1	1	1	1
R	2	10	3	3	3
S	2	8	1	5	4
T	2	4	2	2	3
V	2	5	5	4	3
W	6	1	2	2	3
Y	5	1	1	1	1
# Sequenced	54	64	62	59	57

b.

	Normalized				
	19	23	30	71	82
A	0.86	0.73	0.50	2.98	1.12
C	2.37	0.50	1.55	1.08	2.81
D	1.78	0.50	1.03	0.54	0.56
E	1.78	1.00	0.00	1.08	2.25
F	0.00	0.50	1.55	0.54	1.12
G	0.89	1.00	2.06	1.63	1.96
H	4.74	1.50	1.55	1.08	0.00
I	0.59	1.00	2.06	0.54	1.12
K	1.78	0.00	0.52	0.00	0.00
L	0.20	1.00	2.06	1.08	1.31
M	2.37	1.50	1.55	1.08	1.68
N	0.59	2.00	1.55	0.54	0.56
P	0.00	0.75	0.77	1.36	0.84
Q	0.59	0.50	0.52	0.54	0.56
R	0.40	1.67	0.52	0.54	0.56
S	0.40	1.33	0.17	0.90	0.75
T	0.59	1.00	0.52	0.54	0.84
V	0.59	1.25	1.29	1.08	0.84
W	3.56	0.50	1.03	1.08	1.68
Y	2.96	0.50	0.52	0.54	0.56

$$Normalized = \frac{\%\,found}{\%\,expected} = \frac{\#\ of\ sequences\ present \div \#\ of\ colonies\ sequenced}{\#\ of\ codons\ for\ this\ aa \div \#\ of\ total\ codons\ (32)}$$

Figure S2. Raw data for the structural tolerance of S-824. Following NNK mutagenesis at residues 19, 23, 30, 71, and 82, the ampicillin-resistant population was sequenced to determine the encoded amino acids. (a) Number of occurrences of each amino acid. Green: present in the population. Gray: Not

found. Dark green: Original residue found at this location in the parental S-824 scaffold. (b) Heat map of favored and disfavored amino acids. Using the below equation, the raw colony counts in (a) were converted into a ratio of percent found to percent expected. A value greater than 1 means that an amino acid was more prevalent than expected in a random distribution (blue) and a value less than 1 means that an amino acid was disfavored (red). For example, lysine (K) was disfavored at all but one of the core positions tested; it was not included in the NDT codon used in library design of the Catalytic/Core regions. Out of the hydrophobic residues included in the NDT codon, phenylalanine (F) was the most heavily disfavored as opposed to leucine (L), isoleucine (I), and valine (V). Phenylalanine was therefore encoded at a lower proportion (3%) than the other hydrophobic amino acids at these locations (See Figure S3).

%	CatCor	LpFor_1	LpFor_2
Phe (F)	3	0	0
Leu (L)	19.8	0	0
Ile (I)	18	0	0
Met (M)	0	0	0
Val (V)	19.2	0	0
Ser (S)	4.5	17.6	16
Pro (P)	0	0	0
Thr (T)	0	0	0
Ala (A)	0	0	0
Tyr (Y)	1.25	0	0
His (H)	8.25	1.6	0
Gln (Q)	0	0	0
Asn (N)	7.5	4.4	4
Lys (K)	0	0	0
Asp (D)	8	14	16
Glu (E)	0	0	0
Cys (C)	0.75	0	0
Trp (W)	0	0	0
Arg (R)	4.95	6.4	0
Gly (G)	4.8	56	64
Stop	0	0	0
Sum	100	100	100

Figure S3. Amino acid percentages for variable codons. The second column shows the breakdown for the CatCor residues specified by an NDT codon, with included amino acids highlighted in red. The same specifics are provided for LpFor_1 (VRC, dark blue) and LpFor_2 (RRC, light blue).

Plasmid and Oligonucleotide Sequences Used in This Study

Highlighted = Restriction Sites BseRI NdeI BlpI BsrGI
Antibiotic resistance camR
Lactose operon repressor LacI
Red Letters = p3GLAR ForII and Rev primers

> p3GLAR (p3GLA modified to have correct RBS)

CTAAGAAACCATTATTATCATGACATTAACCTATAAAAATAGGCGTATCACGAGGCCCTTT
CGTCTTCACC*TCGAGAAATCATAAAAAATTTATTTGCTTTGTGAGCGGATAACAATTATAATAG*
*ATTCAATTGTGAGCGGATAACAATTTCACACAGAATTC*ATTAAAGAGGAGAAATTACATATGT
TGTGTTTTACAGTATTATGTAGTCTGTTTTTTATGCAAAATCTAATTTAATATATTGATATTTA
TATCATTTTACGTTTCTCGTTCAGCTTTTTTATACTAAGTTGGCATTATAAAAAAGCATTGCTT
ATCAATTTGTTGCAACGAACAGGTCACTATCAGTCAAAATAAAATCATTATTTGATTTCAAT
TTTGTCCCACTCCCTGCCTCTGTCATCACGATACTGTGATGCCATGGTGTCCGACTTATGCCC
GAGAAGATGTTGAGCAAACTTATCGCTTATCTGCTTCTCATAGAGTCTTGCAGACAAACTGC
GCAACTCGTGAAAGGTAGGC**GGATCC**CCTTCGAAGGAAAGACCTGATGCTTTTCGTGCGCG
CATAAAATACCTTGATACTGTGCCGGATGAAAGCGGTTCGCGACGAGTAGATGCAATTATG
GTTTCTCCGCCAAGAATCTCTTTGCATTTATCAAGTGTTTCCTTCATTGATATTCCGAGAGCA
TCAATATGCAATGCTGTTGGGATGGCAATTTTTACGCCTGTTTTGCTTTGCTCGACATAAAG
ATATCCATCTACGATATCAGACCACTTCATTTCGCATAAATCACCAACTCGTTGCCCGGTAA
CAACAGCCAGTTCCATTGCAAGTCTGAGCCAACATGGTGATGATTCTGCTGCTTGATAAATT
TTCAGGTATTCGTCAGCCGTAAGTCTTGATCTCCTTACCTCTGATTTTGCTGCGCGAGTGGCA
GCGACATGGTTTGTTGTTATATGGCCTTCAGCTATTGCCTCTCGGAATGCATCGCTCAGTGTT
GATCTGATTAACTTGGCTGACGCCGCCTTGCCCTCGTCTATGTATCCATTGAGCATTGCCGC
AATTTCTTTTGTGGTGATGTCTTCAAGTGGAGCATCAGGCAGACCCCTCCTTATTGCTTTAAT
TTTGCTCATGTAATTTATGAGTGTCTTCTGCTTGATTCCTCTGCTGGCCAGGATTTTTTCGTAG
CGATCAAGCCATGAATGTAACGTAACGGAATTATCACTGTTGATTCTCGCTGTCAGAGGCTT
GTGTTTGTGTCCTGAAAATAACTCAATGTTGGCCTGTATAGCTTCAGTGATTGCGATTCGCCT
GTCTCTGCCTAATCCAAACTCTTTACCCGTCCTTGGGTCCCTGTAGCAGTAATATCCATTGTT
TCTTATATAAAGGTTAGGGGGTAAATCCCGGCGCTCATGACTTCGCCTTCTTCCCATTTCTG
ATCCTCTTCAAAAGGCCACCTGTTACTGGTCGATTTAAGTCAACCTTTACCGCTGATTCGTG
GAACAGATACTCTCTTCCATCCTTAACCGGAGGTGGGAATATCCTGCATTCCCGAACCCATC
GACGAACTGTTTCAAGGCTTCTTGGACGTCGCTGGCGTGCGTTCCACTCCTGAAGTGTCAAG
TACATCGCAAAGTCTCCGCAATTACACGCAAGAAAAAACCGCCATCAGGCGGCTTGGTGTT
CTTTCAGTTCTTCAATTCGAATATTGGTTACGTCTGCATGTGCTATCTGCGCCCATATCATCC
AGTGGTCGTAGCAGTCGTTGATGTTCTCCGCTTCGATAACTCTGTTGAATGGCTCTCCATTCC
ATTCTCCTGTGACTCGGAAGTGCATTTATCATCTCCATAAAACAAAACCCGCCGTAGCGAG
TTCAGATAAAATAAATCCCCGCGAGTGCGAGGATTGTTATGTAATATTGGGTTTAATCATCT
ATATGTTTTGTACATCGCTAACTTAATTAGCTGAGCTTGGACTCCTGTTGATAGATCCAGTA
ATGACCTCAGAACTCCATCTGGATTTGTTCAGAACGCTCGGTTGCCGCCGGGCGTTTTTATT
GGTGAGAATCCAAGCTAGGGGAATTCATCGTGACTGACTGACGATCTGCCTCGCGCGTTTC
GGTGATGACGGTGAAAACCTCTGACACATGCAGCTCCCGGAGACGGTCACAGCTTGTCTGT
AAGCGGATGCCGGGAGCAGACAAGCCCGTCAGGGCGCGTCAGCGGGTGTTGGCGGGTGTC
GGGGCGCAGCCATGACCCAGTCACGTAGCGATAGCGGAGTGTATAATTCTTGAAGACGAA
AGGGCCTCGTGATACGCCTATTTTTATAGGTTAATGTCATGATAATAATGGTTTCTTAGAATC
CTGATGTCCGGCGGTGCTTTTGCCGTTACGCACCACCCCGTCAGTAGCTGAACAGGAGGGA
CAGCTGATAGAAACAGAAGCCACTGGAGCACCTCAAAAACACCATCATACACTAAATCAG
TAAGTTGGCAGCATCACCCGACGCACTTTGCGCCGAATAAATACCTGTGACGGAAGATCAC
TTCGCAGAATAAATAAATCCTGGTGTCCCTGTTGATACCGGGAAGCCCTGGGCCAACTTTTG

GCGAAAATGAGACGTTGATCGGCACGTAAGAGGTTCCAACTTTCACCATAATGAAATAAG
ATCACTACCGGGCGTATTTTTTGAGTTATCGAGATTTTCAGGAGCTAAGGAAGCTAAAATG
GAGAAAAAAATCACTGGATATACCACCGTTGATATATCCCAATGGCATCGTAAAGAACATT
TTGAGGCATTTCAGTCAGTTGCTCAATGTACCTATAACCAGACCGTTCAGCTGGATATTACG
GCCTTTTTAAAGACCGTAAAGAAAAATAAGCACAAGTTTTATCCGGCCTTTATTCACATTCT
TGCCCGCCTGATGAATGCTCATCCGGAATTCCGTATGGCAATGAAAGACGGTGAGCTGGTG
ATATGGGATAGTGTTCACCCTTGTTACACCGTTTTCCATGAGCAAACTGAAACGTTTTCATC
GCTCTGGAGTGAATACCACGACGATTTCCGGCAGTTTCTACACATATATTCGCAAGATGTG
GCGTGTTACGGTGAAAACCTGGCCTATTTCCCTAAAGGGTTTATTGAGAATATGTTTTTCGTC
TCAGCCAATCCCTGGGTGAGTTTCACCAGTTTTGATTTAAACGTGGCCAATATGGACAACTT
CTTCGCCCCCGTTTTCACCATGGGCAAATATTATACGCAAGGCGACAAGGTGCTGATGCCG
CTGGCGATTCAGGTTCATCATGCCGTCTGTGATGGCTTCCATGTCGGCAGAATGCTTAATGA
ATTACAACAGTACTGCGATGAGTGGCAGGGCGGGGCGTAATTTTTTTAAGGCAGTTATTGG
TGCCCTTAAACGCCTGGTGCTACGCCTGAATAAGTGATAATAAGCGGATGAATGGCAGAA
ATTCGAAAGCAAATTCGACCCGGTCGTCGGTTCAGGGCAGGGTCGTTAAATAGCCGCTTAT
GTCTATTGCTGGTTTACCGGTTTATTGACTACCGGAAGCAGTGTGACCGTGTGCTTCTCAAA
TGCCTGAGGCCAGTTTGCTCAGGCTCTCCCCGTGGAGGTAATAATTGACGATATGATCATTT
ATTCTGCCTCCCAGAGCCTGATAAAAACGGTTAGGATCGGAGTCAGGCAACTATGGATGAA
CGAAATAGACAGATCGCTGAGATAGGTGCCTCACTGATTAAGCATTGGTAACTGTCAGACC
AAGTTTACTCATATATACTTTAGATTGATTTAAAACTTCATTTTTAATTTAAAAGGATCTAGG
TGAAGATCCTTTTTGATAATCTCATGACCAAAATCCCTTAACGTGAGTTTTCGTTCCACTGA
GCGTCAGACCCCGTAGAAAAGATCAAAGGATCTTCTTGAGATCCTTTTTTTCTGCGCGTAAT
CTGCTGCTTGCAAACAAAAAAACCACCGCTACCAGCGGTGGTTTGTTTGCCGGATCAAGAG
CTACCAACTCTTTTTCCGAAGGTAACTGGCTTCAGCAGAGCGCAGATACCAAATACTGTCCT
TCTAGTGTAGCCGTAGTTAGGCCACCACTTCAAGAACTCTGTAGCACCGCCTACATACCTCG
CTCTGCTAATCCTGTTACCAGTGGCTGCTGCCAGTGGCGATAAGTCGTGTCTTACCGGGTTG
GACTCAAGACGATAGTTACCGGATAAGGCGCAGCGGTCGGGCTGAACGGGGGGTTCGTGC
ACACAGCCCAGCTTGGAGCGAACGACCTACACCGAACTGAGATACCTACAGCGTGAGCTA
TGAGAAAGCGCCACGCTTCCCGAAGGGAGAAAGGCGGACAGGTATCCGGTAAGCGGCAG
GGTCGGAACAGGAGAGCGCACGAGGGAGCTTCCAGGGGGAAACGCCTGGTATCTTTATAG
TCCTGTCGGGTTTCGCCACCTCTGACTTGAGCGTCGATTTTTGTGATGCTCGTCAGGGGGGC
GGAGCCTATGGAAAAACGCCAGCAACGCGGCCTTTTTACGGTTCCTGGCCTTTTGCTGGCCT
TTTGCTCACATGTTCTTTCCTGCGTTATCCCCTGATTCTGTGGATAACCGTATTACCGCCTTTG
AGTGAGCTGATACCGCTCGCCGCAGCCGAACGACCGAGCGCAGCGAGTCAGTGAGCGAGG
AAGCGGAAGAGCGCCTGATGCGGTATTTTCTCCTTACGCATCTGTGCGGTATTTCACACCGC
ATAAATTCCGACACCATCGAATGGTGCAAAACCTTTCGCGGTATGGCATGATAGCGCCCGG
AAGAGAGTCAATTCAGGGTGGTGAATGTGAAACCAGTAACGTTATACGATGTCGCAGAGT
ATGCCGGTGTCTCTTATCAGACCGTTTCCCGCGTGGTGAACCAGGCCAGCCACGTTTCTGCG
AAAACGCGGGAAAAAGTGGAAGCGGCGATGGCGGAGCTGAATTACATTCCCAACCGCGTG
GCACAACAACTGGCGGGCAAACAGTCGTTGCTGATTGGCGTTGCCACCTCCAGTCTGGCCC
TGCACGCGCCGTCGCAAATTGTCGCGGCGATTAAATCTCGCGCCGATCAACTGGGTGCCAG
CGTGGTGGTGTCGATGGTAGAACGAAGCGGCGTCGAAGCCTGTAAAGCGGCGGTGCACAA
TCTTCTCGCGCAACGCGTCAGTGGGCTGATCATTAACTATCCGCTGGATGACCAGGATGCC
ATTGCTGTGGAAGCTGCCTGCACTAATGTTCCGGCGTTATTTCTTGATGTCTCTGACCAGAC
ACCCATCAACAGTATTATTTTCTCCCATGAAGACGGTACGCGACTGGGCGTGGAGCATCTG
GTCGCATTGGGTCACCAGCAAATCGCGCTGTTAGCGGGCCCATTAAGTTCTGTCTCGGCGCG
TCTGCGTCTGGCTGGCTGGCATAAATATCTCACTCGCAATCAAATTCAGCCGATAGCGGAA
CGGGAAGGCGACTGGAGTGCCATGTCCGGTTTTCAACAAACCATGCAAATGCTGAATGAG
GGCATCGTTCCCACTGCGATGCTGGTTGCCAACGATCAGATGGCGCTGGGCGCAATGCGCG

CCATTACCGAGTCCGGGCTGCGCGTTGGTGCGGATATCTCGGTAGTGGGATACGACGATAC
CGAAGACAGCTCATGTTATATCCCGCCGTTAACCACCATCAAACAGGATTTTCGCCTGCTG
GGGCAAACCAGCGTGGACCGCTTGCTGCAACTCTCTCAGGGCCAGGCGGTGAAGGGCAAT
CAGCTGTTGCCCGTCTCACTGGTGAAAAGAAAAACCACCCTGGCGCCCAATACGCAAACC
GCCTCTCCCCGCGCGTTGGCCGATTCATTAATGCAGCTGGCACGACAGGTTTCCCGACTGGA
AAGCGGGCAGTGAGCGCAACGCAATTAATGTGAGTTAGCTCACTCATTAGGCACCCCAGG
CTTTACACTTTATGCTTCCGGCTCGTATGTTGTGTGGAATTGTGAGCGGATAACAATTTCACA
CAGGAAACAGCTATGACCATGATTACGGATTCACTGGCCGTCGTTTTACAACGTCGTGACT
GGGAAAACCCTGGCGTTACCCAACTTAATCGCCTTGCAGCACATCCCCCTTTCGCCAGCTG
GCGTAATAGCGAAGAGGCCCGCACCGATCGCCCTTCCCAACAGTTGC

> S824 DNA sequence

ATGTATGGCAAGTTGAACGACCTGCTGGAAGACTTGCAAGAGGTGCTGAAGAACCTCCAC
AAAAACTGGCACGGTGGCAAAGACAACCTGCACGACGTCGACAACCACTTGCAGAACGTC
ATCGAAGACATCCACGACTTCATGCAAGGCGGTGGCAGCGGCGGCAAGCTGCAAGAGATG
ATGAAAGAGTTCCAACAGGTGTTGGACGAACTCAACAACCACTTGCAAGGCGGTAAACAC
ACCGTGCACCACATCGAACAAAACATCAAAGAGATCTTCCACCACTTGGAAGAGCTTGTA
CATCGCTAA

> Oligonucleotide sequences for *de novo* gene assembly

Mixed bases are indicated according to IDT standards; i.e., (10203040) if the base mixture is 10% A, 20% T, 30% C, and 40% G.

CatCor LpFor_1 *LpFor_2*

Degenerate Oligo 1

GCAGGAAGTGCTGAAGAAC**(34372405)(30001258)T**CATAAAAAC(29106100)(27007300)C(29106100)(27007300)C*(27007300)(27007300)C(27007300)(27007300)C*AAGGATAAC**(34372405)(30001258)T**CATGAT**(34372405)(30001258)T**GATAACCATCTGCAGAACGT

Degenerate Oligo 2

CAGCAGGTGCTGGATGAA**(34372405)(30001258)T**AACAAC(29106100)(27007300)C(29106100)(27007300)C(29106100)(27007300)C*(27007300)(27007300)C(27007300)(27007300)C*AAACAT**(34372405)(30001258)T(34372405)(30001258)T**CATCATATTGAACAGAACATTAAG

Nondegenerate Oligo 1

GTTAACCGGTGAATTGGGCGGGGGTGGCTCCGGAGGCGGCGGCAGCAGCTCTCATATGTGGGCG

Nondegenerate Oligo 2

GTTCTTCAGCACTTCCTGCAGATCTTCCAGCAGATCGTTCAGTTTCGCCCACATATGAGAGCTG

Nondegenerate Oligo 3

TTCATCCAGCACCTGCTGGAATTCTTTCATCATTTCCTGCAGTTTGCCGCCGCTGCCGCCGCCCTGCATAAAATCATGAATATCTTCAATCACGTTCTGCAGATGGTTATC

Nondegenerate Oligo 4

CGAGCCGGATCCTCTATGCACCAGTTCTTCCAGATGATGAAAAATTTCCTTAATGTTCTGTTCAATATGATG

Flanking Primers

For: CAGCAGCTCTCATATGTATG (T_m=56.4°C)

Rev: CTGACTGGATCCAATTCTATGCACCAGTTCTTCCA (T_m=56.4°C)
